# An International Prospective Cohort Study of HIV and Zika in Infants and Pregnancy (HIV ZIP): Study Protocol

**DOI:** 10.3389/fgwh.2021.574327

**Published:** 2021-07-02

**Authors:** Ann Aschengrau, Marisa M. Mussi-Pinhata, John Moye, Nahida Chakhtoura, Kunjal Patel, Paige L. Williams, Brad Karalius, Patricia A. Garvie, Dina Monte, Frances Whalen, Jill Lebov, George R. Seage

**Affiliations:** ^1^Department of Epidemiology, Boston University School of Public Health, Boston, MA, United States; ^2^Department of Pediatrics, Ribeirão Preto Medical School, University of São Paulo, São Paulo, Brazil; ^3^Maternal Pediatric Infectious Disease Branch, Eunice Kennedy Shriver National Institute of Child Health and Human Development, National Institutes of Health, Bethesda, MD, United States; ^4^Department of Epidemiology, Harvard T. H. Chan School of Public Health, Boston, MA, United States; ^5^Departments of Biostatistics and Epidemiology, Harvard T. H. Chan School of Public Health, Boston, MA, United States; ^6^Research Department, Children's Diagnostic and Treatment Center, Fort Lauderdale, FL, United States; ^7^Westat, Rockville, MD, United States; ^8^Research Triangle Institute International, Research Triangle Park, NC, United States

**Keywords:** Zika virus, human immunodeficiency virus, pregnancy, infants, adverse outcomes

## Abstract

Zika virus (ZIKV) infection may adversely affect pregnancies of women living with HIV (WLHIV). Because no study to date has focused on maternal and child effects of HIV and ZIKV co-infection in pregnant women, we undertook the International Prospective Cohort Study of HIV and Zika in Infants and Pregnancy (HIV ZIP). The aims of this two-phase study of pregnant women and their infants are to compare the incidence of ZIKV infection among pregnant women with and without HIV infection and to determine the risk of adverse maternal and child outcomes associated with ZIKV/HIV co-infection at clinical sites in Brazil, Puerto Rico, and the continental United States. Phase I was designed to enroll pregnant women/infant pairs who were: (1) infected with HIV only, (2) infected with ZIKV only, (3) infected with HIV and ZIKV, and (4) not infected with either HIV or ZIKV. A key goal of this phase was to assess the feasibility of enrolling 200 women/infant pairs within a year, with a target of 150 WLHIV, 50 HIV-uninfected women, and a minimum of 20 who were co-infected with HIV and ZIKV. If the feasibility of Phase I proved successful, Phase II would enroll up to 1,800 additional pregnant women/infant pairs to the same four groups. Enrolled women in both phases were to be followed throughout their pregnancy and up to 6 weeks post-partum. Infants were also to be followed for 1 year after birth. To date, Phase 1 data collection and follow-up have been completed. Delineation of possible harmful effects of HIV/ZIKV co-infection will allow the formulation of standard-of-care recommendations to minimize adverse effects but enable the continuation of preventive HIV therapy. Furthermore, while the prospective HIV ZIP study was developed before the COVID pandemic, it is especially relevant today since it can be easily adapted to provide critically important information on the impact of COVID-19 infection or other still unrecognized new agents among pregnant women and their offspring worldwide.

## Introduction

Zika virus (ZIKV) infection may adversely affect pregnancies of women living with HIV (WLHIV). Thus far, there is no study focusing on both maternal and child effects of HIV and ZIKV co-infection in pregnant women. Thus, we undertook the International Prospective Cohort Study of HIV and Zika in Infants and Pregnancy (HIV ZIP) to investigate these possible effects among WLHIV in Brazil, Puerto Rico and the United States. This study is especially relevant given the current COVID-19 pandemic since the World Health Organization (WHO) has not yet developed a protocol for studying the impact on pregnant women who are infected with COVID-19 alone or co-infected with other microorganisms.

### History and Epidemiology of ZIKV

ZIKV, a vector-borne single-stranded RNA virus of the genus *Flaviviridae*, was first identified in rhesus macaques in the Zika forest, Uganda in 1947. Between its first isolation in monkeys until 2007, reports of human cases were rare and sporadic, the infection was largely asymptomatic and no epidemics were observed. In 2007, an outbreak of ZIKV occurred in the Federated States of Micronesia (1) and in 2013 another outbreak occurred in French Polynesia (2).

In May 2015, the first cases of ZIKV in Brazil were reported in the northeast part of the country. The virus subsequently spread throughout South America, the Caribbean, and Puerto Rico. By December 2016, 48 countries in the Americas had reported vector-borne transmission of ZIKV (3). However, as of July 2019, this expanded to 87 countries and territories worldwide with evidence of autochthonous mosquito-borne transmission of ZIKV, distributed across four of the six WHO Regions, including Africa, the Americas, South-East Asia, and the Western Pacific (4).

With introduction of ZIKV, Brazil witnessed an epidemic of microcephaly cases among newborns. According to the most recent Pan American Health Organization (PAHO) report, 14,558 suspected cases of microcephaly and other central nervous system (CNS) malformations were reported in Brazil during 2015–2017 as compared to an annual average of 163 cases of microcephaly during 2001–2014 (5). From 2015 to 2017, 27 countries and territories in the Americas (including the U.S. and Puerto Rico) reported confirmed cases of birth defects and other abnormalities associated with ZIKV infection (3).

### Clinical Sequelae of ZIKV Infection During Pregnancy

Microcephaly was not systematically examined and reported following ZIKV infection before the outbreak in the Americas. Besnard et al. retrospectively reported a summary of the 2013–2014 ZIKV outbreak in French Polynesia (6). They described ten cases of congenital cerebral malformations in fetuses and newborns including eight with major brain lesions and severe microcephaly.

In January 2016, one of the first reports of microcephaly cases in the Brazilian epidemic was published (7). All 35 infants met the case definition of a head circumference > 2 standard deviations below the mean for sex and gestational age, and 74% of their mothers reported a rash-like illness during pregnancy. Additional case series have been reported linking prenatal ZIKV infection to an increasing number of abnormalities in infants including joint contractures, clubfoot, and hearing loss (8, 9).

Formal epidemiological studies found further evidence of adverse birth and pregnancy outcomes associated with prenatal ZIKV infection. An initial Brazilian study of symptomatic pregnant women found that 46% of ZIKV-exposed pregnancies vs. 11.5% of unexposed pregnancies had adverse pregnancy and infant outcomes, including miscarriage, fetal death, growth retardation, microcephaly and other CNS abnormalities (10). Findings from a case-control study in Brazil detected a strong association of maternal ZIKV infection with microcephaly and brain abnormalities independent of exposure to other potential teratogens (11, 12). More recently, two large cohorts from the U.S. (13, 14) and one from French territories in the Americas (15) reported smaller estimates (5–7%) of pregnancy losses and/or birth defects potentially related to ZIKV in newborns of symptomatic women. A meta-analysis of studies encompassing 2,941 ZIKV-exposed pregnancies reported a 2.7% prevalence of microcephaly among all live births (16).

### HIV Infection in Pregnant Women

Currently, more than 38 million people are living with HIV and 1.7 million new infections occurred in 2018 (17). Although the prevention of mother-to-child transmission (MTCT) of HIV has become a standard of care worldwide, in 2018 there were ~160,000 children infected through this mode (17).

The first cases of Acquired Immune Deficiency Syndrome (AIDS) in Brazil were reported in 1982, and Brazil was one of the first countries to provide universal access to antiretroviral therapy (ART), i.e., since 1996 (18). In 2018, Brazil had a total of 900,000 people living with HIV with 66% on ART (19).

In Brazil, it is estimated that ~0.38% of pregnant women are infected with HIV (20), corresponding to 11,000 HIV-infected women giving birth each year. Since 1990, specialty care, antiretroviral (ARV) drugs, and formula to feed the babies have been available for free to these women to avoid MTCT. Recently, a nationwide study estimated that 95% of these women had at least one appointment for prenatal care and the vertical transmission rate decreased to 2% (21). By comparison, in the U.S., <5,000 WLHIV give birth each year. With the combination of routine HIV testing of pregnant women and access to ART, the MTCT rate has dropped below 1% (22). While the use of ART during pregnancy has demonstrated a tremendous public health success, concerns remain regarding both the short-term effects of HIV and ART during pregnancy (23) and the longer-term effects among children who were HIV-exposed *in utero* but uninfected (HEU) at birth. A number of possible toxicities have been reported in ART-exposed children with some of the major areas being growth (including microcephaly), neurobehavioral development, and mitochondrial dysfunction (24–28).

### Consequences of Prenatal HIV Infection on Immunity and Impacts of Co-infections on Mother and Fetus

To promote and support pregnancy and the growing fetus, an immunological adaptation is needed. This sanctuary is damaged when this inherent placental protection is broken by a viral infection. Adverse outcomes may result, including increased maternal morbidity related to the infection, propagation of other microorganisms, fetal demise, pre-term labor, and MTCT of other infectious agents (29).

HIV infection is known to have deleterious effects on the immune system, mainly on the CD4+ T-cell compartment, B-lymphocytes and antibody responses to pathogens and vaccines. These alterations are linked to immune activation/dysregulation and chronic inflammation (30). Even after treatment, HIV infection evolves into a chronic condition with the potential to continuously affect the host immune system (31). These facts are especially important to the pregnant mother and fetus when the pregnancy is threatened by a superimposed latent or acutely acquired infection.

Increased risk of severe illness during pregnancy from viral infections has been reported during pandemics of influenza, Ebola, and Lassa fever, even in HIV- uninfected women (32). With regards to the effect of the past ZIKV outbreak on pregnant WLHIV, there was a case report of an immunosuppressed pregnant woman on a combination ART (cART) regimen who presented with a mild disease (33). While the pregnant woman had a rapid and complete recovery, her severely affected fetus died at 20 weeks' gestation (33). Considering the potential for more severe ZIKV disease in pregnant WLHIV, it is very relevant to examine pregnant women with both ZIKV and HIV infections to assess the consequences of co-infection on both mother and infant health.

Moreover, it is possible that ZIKV may act as an HIV facilitator pathogen. That is, it could enhance HIV replication during infection due to the release of cytokines that can activate CD4+T-cells, or by directly binding to HIV proteins that support HIV replication (34). High maternal HIV load is the strongest risk factor for MTCT of HIV, and reduction of HIV load with ART significantly reduces risk (35). Possibly, any infection that increases HIV load in plasma, the genital tract or breast milk may indirectly raise the risk of MTCT. In addition, HIV's systemic immunological activation may impair the placental barrier and boost the risk of MTCT of the infecting agent, including ZIKV.

In contrast to HIV, the correlates of MTCT of ZIKV and the fetal disease caused by ZIKV infection are currently unknown. However, it is likely that the immune system dysfunction of HIV-infected pregnant women will potentially be enhanced by acute viral infection, even in the presence of ART, increasing the risk of MTCT of both HIV and ZIKV and their consequences.

### Rationale for HIV ZIP Study

While remarkable strides have been made in the treatment of pregnant WLHIV over the last decade, resulting in immune restoration for their own health and the prevention of MTCT, the occurrence of ZIKV infection among pregnant WLHIV raises serious concerns regarding the ability of ART to suppress HIV RNA levels and prevent MTCT of both viruses. There is also a growing number of significant adverse infant outcomes, particularly those related to the CNS, following prenatal ZIKV infection (36), and because HIV is also a neurotropic virus, the ability of a pregnant WLHIV to maintain HIV RNA suppression will be critical for her child's health. Therefore, we have undertaken a prospective cohort study to investigate both maternal and child effects of HIV and ZIKV co-infection in pregnant women. Delineation of adverse effects will allow formulation of standard-of-care recommendations to minimize adverse effects but enable continuation of preventive therapy.

While rarely conducted, prospective studies such as HIV ZIP are poised to provide critically important information in the midst of an epidemic (37). As we are currently experiencing another widespread pandemic stemming from COVID-19 (38), the need for rapid deployment of studies with adaptable protocols is even more urgent.

## Methods

### Study Design and Setting

The HIV ZIP Study is a two-phase international prospective cohort study of pregnant women and their infants whose goals are to compare the incidence of ZIKV infection among pregnant women with and without HIV infection, and to determine the risk of adverse maternal and child outcomes associated with ZIKV/HIV co-infection at clinical sites in Brazil, Puerto Rico, and the continental U.S.

Phase I was designed to enroll HIV-infected and -uninfected pregnant women with or at risk for Zika infection. In particular, it was designed to enroll four groups of women who were: (1) infected with HIV only, (2) infected with ZIKV only, (3) infected with HIV and ZIKV, and (4) not infected with either HIV or ZIKV. A key goal of this Phase was to assess the feasibility of enrolling within 1 year 200 pregnant women with or at risk of ZIKA infection, with a target of 150 WLHIV, 50 HIV-uninfected women, and a minimum of 20 who were co-infected with HIV and ZIKV. Should the feasibility of Phase I prove successful, Phase II would enroll up to 1,800 additional pregnant women to the four groups described above. In both phases, enrolled women were to be followed throughout their pregnancy and up to 6 weeks post-partum. Infants born to enrolled women were also to be followed for a year after birth. As of this writing, enrollment, data collection and follow-up for Phase 1 of the study have been completed.

### Eligibility Criteria

The study enrolled pregnant women who were: (1) aged 15 years and older, (2) resided in geographic areas accessible to participating clinical research sites in Brazil, Puerto Rico, U.S. states Florida, New York and Texas, (3) <18 weeks' gestation, (4) either HIV-infected or uninfected (mainland U.S. sites only) as per laboratory confirmation, and (5) met criteria for ZIKV infection risk. The latter criteria included at least one of the following: (1) has resided for at least 3 months or traveled within the last 3 months to a country or U.S. Territory with active cautionary, or previously active or cautionary ZIKV transmission designation, (2) sexual partner has resided in or traveled within the last 6 months to a country or U.S. Territory with active, cautionary, or previously active or cautionary ZIKV transmission designation, or was diagnosed with ZIKV within the previous 6 months; or (3) household member has been diagnosed with ZIKV infection or has traveled since the woman's last menstrual period to a country or U.S. Territory with active cautionary, or previously active or cautionary ZIKV transmission designation. HIV-uninfected women were not enrolled in Brazil or Puerto Rico because the Zika in Infants and Pregnancy (ZIP) Study investigators provided all necessary data on their HIV-uninfected participants *via* a data sharing agreement.

Although we want to enroll women at risk for ZIKV infection as early in pregnancy as possible and follow them throughout pregnancy, to maximize opportunities for enrolling ZIKV-infected women, the study also allowed enrollment of pregnant women at 18 weeks or greater gestation who presented with acute ZIKV-like symptoms with confirmed ZIKV infection by a positive ZIKV-RNA detection test at the screening visit. Lastly, all newborns were also enrolled in this study upon parents' or legal guardians' consent.

### Study Objectives

The primary aims of HIV ZIP study are:

#### Phase I

- To determine the feasibility of enrolling pregnant women with ZIKV/HIV co-infection, HIV infection alone, ZIKV infection alone, and women with neither ZIKV or HIV into a prospective cohort study at clinical sites in Brazil, Puerto Rico, and the continental United States.

#### Phase II

1. To compare HIV viral suppression in WLHIV with and without ZIKV co-infection during pregnancy and the time of delivery.

- To compare the incidence of ZIKV infection among pregnant women with and without HIV infection.
- To compare the incidence of adverse pregnancy outcomes between women co-infected with HIV and ZIKV, women infected with either HIV or ZIKV alone, and women without either infection.
- To compare the incidence of vertical transmission of HIV and ZIKV between women co-infected with HIV and ZIKV, and women infected with either HIV or ZIKV alone.
- To compare the incidence of congenital malformations and other adverse outcomes (including microcephaly, neonatal death, central nervous system malformations, and ocular abnormalities) among offspring of women co-infected with HIV and ZIKV, women infected with either HIV or ZIKV alone, and women without either infection.
- To compare the long-term effects on growth, hearing, vision and neurodevelopment of co-infection among children with *in utero* exposure to HIV and ZIKV, *in utero* exposure to either HIV or ZIKV alone, and no *in utero* exposure to either virus.

The secondary aims are to:

- To compare the severity of maternal and infant outcomes of ZIKV infection in symptomatic and asymptomatic women.
- To determine if co-infections and cofactors including social and environmental factors contribute to the presence of CNS malformations and if these factors influence the severity of adverse outcome in the offspring.

### Data Collection for Phases 1 and 2

#### Maternal Participants

Study visits occurred once per trimester during pregnancy, at delivery and ~6 weeks post-partum. At baseline, demographic characteristics were collected including the city of residence, education, occupation (self/partner), household characteristics (e.g., city/suburb/ rural, number of windows/doors, sanitation methods, presence of animals, smoking and substance use, and environmental exposures such as pesticides). A targeted maternal physical examination was also conducted which included general appearance and an assessment of signs and symptoms of acute ZIKV infection such as fever, rash, arthralgia, myalgia, pruritus, headache, eye pain, conjunctivitis, and lymphadenopathy.

Baseline and follow-up medical, medication, substance use and other risk factor history were obtained by interview and medical record abstraction. Medical history included a recent history of symptoms consistent with ZIKV infection in the women, their sexual partners and household members. Fetal ultrasounds (US) were performed (or data abstracted from clinically performed US) in the first, second and third trimesters.

At each visit, WLHIV had all available HIV viral loads (VLs) and CD4+/CD8+ absolute T-cells counts, and percentages abstracted from their medical records. If these laboratory results were unavailable, blood samples were collected for these laboratory assessments. Testing of urine and blood was performed at each visit for the diagnosis of ZIKV infection (anti-ZIKV IgM Abs, and, if indicated, ZIKV RNA). Due to the cross-reactivity between assays detecting antibodies against ZIKV and Dengue virus, for those participants with a history of dengue virus (DENV) infection or living in a DENV endemic region, testing for DENV IgM Abs, DENV IgG Abs, and DENV NS1 Ag was also done if ZIKV IgM Abs result was positive/equivocal and ZIKV rRT-PCR negative. Medical histories were reviewed for results of clinical laboratory tests for co-infections such as toxoplasmosis, rubella, cytomegalovirus (CMV), herpes simplex virus, chikungunya, yellow fever, West Nile virus, and sexually transmitted diseases. Plasma and urine samples from all visits, and cord blood samples and placental tissue collected at delivery were stored for future testing. If a decision is made to proceed with Phase 2, we will also test women for COVID-19. The process for doing so will be determined at that time. See Appendix I for the complete schedule of maternal evaluations.

#### Infant Participants

Shortly after birth, data were collected on the infant's demographic and delivery characteristics such as location of birth, mode of delivery, single or multiple, gestational age, and results of a physical examination that included birth weight, anthropometric measurements including head circumference, Apgar scores, and neurological, hearing and ophthalmologic assessments. Physical exams were conducted at birth and at three, 6 and 12 months of age. These exams assessed growth parameters, including head circumference, and included hearing and ophthalmologic evaluations. If initial hearing and/or ophthalmology screenings were abnormal, referral for additional testing was made as per local standard of care. Results of any imaging assessments done for clinical care were recorded. All ZIKV-exposed infants were to have a cranial ultrasound within the first 3 months. During the first year of life, standardized infant neurodevelopmental screening assessments were conducted to grossly evaluate cognitive, receptive and expressive language, fine and gross motor development markers and developmental milestones using the Bayley Scales of Infant and Toddler Development®, Third Edition (BSID®-III) Screening Test (English and Portuguese speaking sites) (39) or the Ages and Stages Questionnaires®, Third Edition (40) (Spanish speaking sites only due to the BSID®-III Screening Test being unavailable in Spanish). Upon evidence of neurodevelopmental screen failure, infants were provided a standardized comprehensive evaluation of the same domains *via* the Complete BSID®-III (all languages/all sites) (41) to more accurately identify potential neurodevelopmental risk and/or deficits, including appropriate clinical referrals (i.e., neurology). Any additional tests were performed according to the local standard of care. The results of these tests were recorded on the study case report forms.

Urine and peripheral blood tests were performed at birth and subsequent visits for the diagnosis of ZIKV infection. Testing for other infections such as TORCH and DENV may also be performed, if indicated. HIV-related testing results such as CD4+ T-cells and HIV-RNA done as per standard of care were abstracted from medical records. If a decision is made to proceed with Phase 2, infants born to COVID-19-positive mothers will also be tested for this virus. See Appendix II for the complete schedule of infant evaluations.

### Data Management

Westat, in collaboration with the HIV ZIP Protocol Team, has been responsible for this study. All Case Report Forms (CRFs) are available for download from the HIV ZIP link on the NICHD Clinical Studies website (www.nichdclinicalstudies.org). Data collection has been the responsibility of the research staff at the sites under the supervision of the site investigators who are responsible for ensuring the accuracy, completeness, legibility, timeliness, and documentation of the study data. Data are entered and managed in a secure 21 Code of Federal Regulations (CFR) Part 11-compliant electronic data capture (EDC) system called REDCap Cloud (RCC). Access to the RCC is limited to approved individuals. Clinical research records are being stored by the participating sites in a manner that ensures privacy, confidentiality, security and limited accessibility. Records relating to research and IRB/IEC records are required by the HHS regulations 45 CFR Part 46.115(b) to be retained for at least 3 years after completion of the research.

### Data Analysis Plan

The following data analysis plan addresses the primary aims of the study.

#### Phase I Aim

To determine the feasibility of enrolling pregnant women with ZIKV/HIV co-infection, HIV infection alone, ZIKV infection alone, and women without either infection.

Feasibility in enrolling a total of 200 pregnant women within a year, with a target of 150 WLHIV across all sites, 50 HIV-uninfected women sites in the continental United States only, and a minimum of 20 women who are co-infected with HIV and ZIKV will be assessed by monitoring total accrual, accrual by study sites and accrual into the four study groups. If the feasibility phase proves successful, enrollment into Phase II will begin with the goal of enrolling up to 1,800 pregnant women at risk for ZIKV, bringing to total target enrollment to 2,000.

#### Phase II Aims

##### Aim 1

To compare HIV viral suppression in WLHIV with and without ZIKV co-infection during pregnancy and at delivery.

To address this aim, the study population will be restricted to pregnant WLHIV. Lack of HIV viral suppression (e.g., “unsuppressed VL”) will be defined as having a VL > 1,000 copies/mL as this is the VL threshold identified with HIV transmission risk. HIV viral suppression will be assessed at four time points—start of first, second and third trimesters and at the time of delivery. The proportion with unsuppressed VL at these time points will be compared by presence of ZIKV co-infection at these same time points using Fisher's exact-tests. Logistic regression models will be used to estimate odds ratios (ORs) and 95% Confidence Intervals (CIs) for lack of HIV viral suppression at these time points comparing pregnant WLHIV with ZIKV co-infection to those without ZIKV co-infection, adjusting for potential confounders. We hypothesize that pregnant WLHIV with ZIKV co-infection will have a higher risk of having unsuppressed VL during pregnancy and at the time of delivery compared to those without ZIKV infection.

##### Aim 2

To compare the incidence of ZIKV infection among pregnant women with HIV infection and those without HIV infection.

To address this aim, the study population will be restricted to women without the presence of ZIKV infection at enrollment. Cumulative incidence of ZIKV infection will be calculated as the number of new confirmed ZIKV infections identified by the end of pregnancy divided by the total number of completed pregnancies, overall and by HIV status. The relative risk and 95% CI for ZIKV infection comparing WLHIV to HIV-uninfected women will be estimated using log-binomial regression, adjusting for potential confounders.

##### Aim 3

To compare the incidence of adverse pregnancy outcomes between women co-infected with HIV and ZIKV, women infected with either HIV or ZIKV alone and doubly uninfected women.

The cumulative incidence of adverse pregnancy outcomes (i.e., miscarriage, stillbirth and preterm birth) will be calculated as the number of adverse outcomes identified by the end of pregnancy divided by the total number of completed pregnancies, overall and by HIV/ZIKV status. The relative risk and 95% CI for adverse pregnancy outcome comparing women who are HIV and ZIKV co-infected, HIV-infected only and ZIKV-infected only to women without either infection will be estimated using log- binomial regression, adjusting for potential confounders. Potential confounders include demographic and socioeconomic status variables, smoking, alcoholic beverage consumption and substance use during pregnancy and exposure to specific environmental contaminants during pregnancy.

##### Aim 4

To compare the incidence of mother to child transmission (MTCT) of HIV and ZIKV between women co-infected with HIV and ZIKV, and women infected with either HIV or ZIKV alone.

To address this aim, the study population will be restricted to women who are infected with HIV, ZIKV or both. Among the pregnant women who are doubly infected, we will estimate the cumulative incidence of MTCT of both HIV and ZIKV, HIV only, and ZIKV only. Among pregnant women who are HIV-infected only, we will estimate the risk of MTCT of HIV. Among pregnant women who were ZIKV-infected only, we will estimate the risk of perinatal ZIKV transmission. We will compare the risk of MTCT of HIV among women who were doubly infected to women only infected with HIV using log-binomial regression, adjusting for potential confounders. Similarly, we will compare the risk of MTCT of ZIKV among women who were doubly infected to women only infected with ZIKV using log- binomial regression, adjusted for potential confounders.

##### Aim 5

To compare the incidence of congenital malformations and other adverse outcomes (including microcephaly, neonatal death, CNS malformations, and ocular abnormalities) among offspring of women co-infected with HIV and ZIKV, women infected with either HIV or ZIKV alone and doubly uninfected women.

The cumulative incidence of congenital malformations and other adverse outcomes among infants will be calculated overall and by perinatal HIV/ZIKV exposure status. The risk ratios and 95% CI for adverse infant outcomes comparing women who were HIV and ZIKV co-infected, HIV-infected only and ZIKV-infected only to doubly uninfected women will be estimated using log-binomial regression, adjusting for potential confounders. Potential confounders include demographic and socioeconomic status variables, substance use during pregnancy and exposure to specific environmental contaminants during pregnancy.

##### Aim 6

To compare the long-term effects on growth, hearing, vision and neurodevelopment (ND) among children with *in utero* exposure to HIV and ZIKV, *in utero* exposure to either HIV or ZIKV alone, and no *in utero* exposure to either virus.

The incidence rate of long-term outcomes among infants will be calculated as the number of adverse outcomes identified over follow-up divided by the total person- time of follow-up, overall and by perinatal HIV/ZIKV exposure status. The incidence rate ratios and 95% CI for adverse infant outcomes comparing women who were doubly exposed, HIV-exposed only, and ZIKV-exposed only to women without either infection will be estimated using Poisson regression, adjusting for potential confounders. Potential confounders include demographic or socioeconomic status variables, substance use during pregnancy and exposure to specific environmental contaminants during pregnancy.

### Power and Sample Size Considerations

Although the key goal of Phase I is to assess feasibility of enrolling WLHIV and HIV-uninfected women with or without ZIKV infection, it should be noted that the proposed sample size of 150 WLHIV will provide 80% power to detect a difference in percent with unsuppressed VL of 38.7 vs. 10% between WLHIV with ZIKV and WLHIV without ZIKV infection, assuming 10% of the WLHIV enrolled (and meeting ZIKV risk criteria) have ZIKV infection by delivery.

In the full Phase II study, we anticipated enrolling an additional 1,800 pregnant women to bring total enrollment to 2,000 women. This large enrollment allows precise estimates of event rates and sufficient power to compare subgroups defined on the basis of prenatal HIV/ZIKV exposure or other characteristics. For the target enrollment of 2,000 women, we will be able to estimate an overall event rate of 10% with a precision (i.e., 1.96^*^ s.e.) of ± 1.3%. If 1,000 of these 2,000 women are HIV-infected, then an estimated 10% percent with ZIKV infection within this subgroup would have a precision of 1.8%. Even within a smaller subgroup of 500 participants, we would have precision levels ranging from 2.0 to 3.6% for corresponding event rates of 5–20%. In designing the study, we considered power and detectable differences at interim enrollments of 500 and 1,000 women, but for simplicity have provided power and/or detectable differences for the remaining aims only for the full target sample size of 2,000.

For comparison of the percent with unsuppressed VL in Aim 1 of Phase 2, our power is based only on the WLHIV enrolled. Table 1 below summarizes the minimum detectable differences in proportions with suppressed VL by the end of pregnancy (and corresponding ORs) between WLHIV who were ZIKV-exposed as compared to WLHIV who were ZIKV-unexposed, assuming that 60% (*N* = 1,200) of the full target sample size of 2,000 women is HIV-infected. For example, the study design provides 80% power to detect a difference in the percent with unsuppressed VL of 16.7 vs. 10% comparing women who were ZIKV-infected vs. ZIKV-uninfected, assuming that 20% of the 1,200 become ZIKV-infected by the end of their pregnancy, corresponding to an OR equal to 1.80. The range of 10–20% assumed for percent with unsuppressed HIV VL was based on data from the Pediatric HIV/AIDS Cohort Study (PHACS) Surveillance Monitoring for ART Toxicities (SMARTT) study of pregnant WLHIV who are presumed to be ZIKV-uninfected. If the percent with ZIKV infection increases to 50%, the minimum detectable OR at 80% power decreases to 1.64 reflecting a difference between 15.4 vs. 10%, or an absolute difference of 5.4% in unsuppressed VL. Higher rates of unsuppressed VL (20%) among the ZIKV-uninfected will also translate to smaller detectable ORs, with absolute differences in percent with unsuppressed VL ranging from 7 to 9%.

**Table 1:** Minimum detectable odds ratios (ORs) (and percent with unsuppressed VL among ZIKV- infected) at 80% power comparing women who were HIV+/ZIKV+ vs. HIV+/ZIKV-.

**% ZIKV-infected**	**Minimum detectable ORs (% with unsuppressed VL in ZIKV+) given assumed percent with unsuppressed VL in ZIKV-uninfected:**	
	**10%**	**20%**
20%	1.80 (16.7%)	1.60 (28.5%)
30%	1.69 (15.8%)	1.51 (27.4%)
40%	1.65 (15.5%)	1.48 (27.0%)
50%	1.64 (15.4%)	1.47 (26.8%)
60%	1.66 (15.5%)	1.48 (27.0%)
70%	1.72 (16.0%)	1.52 (27.6%)

For Aim 2 of Phase 2, we provide the minimum detectable OR at 80% power in Table 2 for comparing the percent with ZIKV infection by the end of pregnancy between WLHIV and HIV-uninfected women, based on a final target enrollment of 2,000 women, and assuming the percent with HIV infection among those enrolled is 40% (ORs are similar for percent with HIV infection ranging from 35 to 65%). Assuming 30% of the HIV-uninfected women become ZIKV-infected by the end of pregnancy, then the study design will provide 80% power to detect a difference in ZIKV infection of 36.0 vs. 30% for WLHIV vs. HIV-uninfected women, or an absolute difference of 6.0% (with corresponding OR = 1.30).

**Table 2:** Minimum detectable odds ratios (ORs) (and percent of ZIKV-infected among HIV-infected) at 80% power for various assumed ZIKV infection rates among HIV-uninfected.

**% with ZIKV infection in the HIV-uninfected group**	**Minimum detectable ORs (% ZIKV+ in HIV-infected) assuming 40% of enrolled are ZIKV-infected**
20%	1.36 (25.3%)
30%	1.31 (36.0%)
40%	1.30 (46.3%)
50%	1.29 (56.4%)
60%	1.30 (66.2%)
70%	1.33 (75.7%)

To address the power for Aim 3 of Phase 2, a number of scenarios for possible percentages of enrollment within the four different subgroups defined by the cross-classification of HIV infection and ZIKV infection were evaluated and the power for comparing the percent of pregnancies with adverse outcomes for each of the first three subgroups to the doubly uninfected group was evaluated for a range of sample sizes (Table 3). From this table, it is evident that the power for detecting an OR of 2 when comparing pairs of subgroups (e.g., HIV+/ZIKV+ vs. HIV-/ZIKV-) is high (>80%) when the background rate of adverse outcomes is at least 10% in the reference group (doubly uninfected). The first two scenarios in Table 3 reflect an assumption that a total of 40% of the women enrolled will be HIV-infected, the second two scenarios assume 50% will be HIV-infected, and the last two scenarios assume 60% will be HIV-infected.

**Table 3:** Power for detecting differences in rate of adverse pregnancy outcomes (for Aims 3 and 5) based on assumed proportion in the HIV and ZIKV co-infected, HIV- infected and ZIKV-infected groups as compared to the HIV and ZIKV-uninfected group.

**Percent of enrolled in HIV+/ZIKV+, HIV+/ZIKV-, HIV-/ZIKV+, and HIV-/ZIKV- groups**	**Power for detecting 2-fold difference in odds of adverse outcomes for HIV+/ZIKV+, HIV+/ZIKV-, and HIV-/ZIKV+** **groups vs. HIV-/ZIKV- group, based on assumed background rates in reference group**		
	**5%**	**10%**	**20%**
12, 28, 12, 48%	66.4, 85.7, 66.4%	89.6, 98.5, 89.6%	98.5, 100, 98.5%
20, 20, 24, 36%	73.7, 73.7, 77.0%	94.2, 94.2, 95.8%	99.6, 99.6, 99.8%
15, 35, 10, 40%	69.5, 84.9, 58.6%	91.8, 98.4, 83.6%	99.1, 100, 96.6%
25, 25, 20, 30%	72.9, 72.9, 69.3%	94.0, 94.0, 92.0%	99.6, 99.6, 99.3%
18, 42, 8, 32%	68.9, 80.8, 49.5%	91.6, 97.3, 74.8%	99.2, 99.9, 92.3%
30, 30, 16, 24%	68.3, 68.3, 59.6%	91.8, 91.8, 85.2%	99.4, 99.4, 97.7%

Aim 4 involves comparing the rate of MTCT of either HIV or ZIKV. This study is not powered to detect differences in the rate of MTCT of HIV, but will provide good precision for the estimation of such rates. Assuming 40% of women enrolled are HIV-infected, the precision (half-width) of CIs is 0.055% under the assumption of a 0.5% HIV transmission rate. If 60% of women are HIV-infected, the precision would increase and CI half widths would correspondingly decrease to 0.045%.

The power calculations for Aim 5 are provided in Table 3 for a range of AE rates in the doubly uninfected group, and under various scenarios for percent of total accrual in each of the four subgroups. As an example, if the underlying percent with congenital malformations or other defects is 10% in the doubly infected group, the target sample size of 2,000 would provide over 90% power for most pairwise comparisons of interest.

Given the lack of information regarding long-term effects of ZIKV infection in any population, and among people living with HIV in particular, Aim 6 is considered exploratory and no power calculations are provided for this specific aim. However, the power calculations in Table 3 can serve as guidance regarding the range of power that would be anticipated for the full study accrual.

### Ethical Standard

The study was conducted in accordance with the World Medical Association's Declaration of Helsinki (42) and performed in accordance with relevant institutional and national guidelines, with prior approval of all relevant institutional ethics committees. The study was also registered in ClinicalTrials.gov (IDNO: NCT03263195). Written informed consent obtained from each study participant or their legally authorized representative.

### Study Strengths and Limitations

As described above, this two-stage study has been designed and powered to achieve the study objectives *via* detecting outcomes differences between four main study groups. The two-stage aspect of the design also enables a cost- and time-efficient assessment of the feasibility of accomplishing these objectives. The extensive data collection which involves gathering multiple biological samples (including placenta samples) over the course of pregnancy is also a strength. Furthermore, the prospective design also makes selection bias, that is selection of study subjects on the basis of *both* their exposure (HIV and ZIKV status) and outcome status (adverse maternal and infant outcomes) highly unlikely. Lastly, data collection included information on numerous confounding variables enabling their control during data analysis.

The study limitations stem mainly from possible misclassification of HIV and ZIKV infection status due to laboratory and other sources of error. If these errors are non-differential (the most likely scenario in a prospective study), the results of the study would be biased toward the null. Misclassification of confounding variables is also possible, particularly for variables that rely on maternal recall. Any misclassification of these variables would lead to residual confounding. Lastly, if measures to minimize loss to follow-up are unsuccessful, missing maternal and infant outcome data could not only reduce the power of the study but also bias the results either toward or away from the null.

## Discussion

ZIKV has widely spread over the world but mainly in the regions of Africa, South America, and Southeast Asia. It disseminated in tropical and subtropical areas that are also endemic with other infections, such as other flavivirus and HIV (43). The co-circulation and co-infection of ZIKV with these viruses represented a biomedical and public health challenge, especially for pregnant women.

The potential facilitation of MTCT of HIV and other viruses in the presence of co-infections with other microorganisms during gestation, even in era of ART, has been investigated in many studies. It has been shown that placental malaria is associated with increased MTCT of HIV, even at low maternal HIV viral loads (44). Syphilis, a common co-infection in WLHIV, can also facilitate *in utero* transmission of HIV to infants (45). Similarly, type 2 herpes simplex virus co-infection has been associated with increased intrapartum HIV transmission (46). Although the Hepatitis B Virus (HBV) infection does not appear to be independently associated with increased transmission of HIV, maternal HIV/HBV co-infection may increase HBV transmission to the infant (35). In addition, mothers with HIV/Hepatitis C Virus (HCV) co-infection are at increased risk for perinatal transmission of both viruses (47). Likewise, the prevalence of congenital CMV infection among infants born to WLHIV is higher (2–7%) as compared to the general newborn population (0.7%) (48). Although there are data suggesting that ART decreases the risk of congenital CMV infection among WLHIV (49), a recent report shows that congenital CMV infection remains higher among infants born to WLHIV receiving ART (50).

The human placenta has a critical role in restricting vertical transmission of microbes. However, the barrier is not perfect once several microorganisms breach it during gestation. Also, the exact mechanisms by which this organ limits microbial transmission from mother to infants are uncertain (51). It has been suggested that infection with one pathogen could predispose to infection with another *via* immune-mediated breakdown of the placenta cells such as syncytium trophoblasts (52). Microbes may also disrupt the placenta integrity affecting the growth of the fetus, independently of the occurrence of fetal infection (32).

Currently, it is unclear whether HIV infection increases the susceptibility to ZIKV infection and whether ZIKV infection worsens HIV infection, particularly during pregnancy. Importantly, two potential interactions between ZIKV and HIV infections in pregnant women could adversely influence maternal health and facilitate vertical transmission of both virus imposing deleterious effects on the fetus and infants: (1) the impact of the ZIKV on the immune system of the mother living with HIV, and (2) the dysfunctional effect of ZIKV on the placenta.

Like other flaviviruses, ZIKV has a broad tropism for many types of cells. Viral RNA has been isolated from human bodily fluids including blood, saliva, urine, tears, aqueous humor, breast milk, semen, and vaginal secretions (53). After replication at the inoculation site and lymphoid tissues, large numbers of mononuclear cells are recruited to amplify the virus. A recent *in vitro* study identified that CD14+ monocytes are the primary cellular target for both the African and Asian lineages of ZIKV in human blood and characterized the heightened sensitivity of monocytes of pregnant women to this virus especially during the early stages of pregnancy (54). Further, it was found that while higher viral burdens were detected in CD14+ monocytes infected with the African-lineage ZIKV strain than in those infected with the Asian-lineage ZIKV strain, the Asian strain largely promoted expansion of non-classical monocytes, the suppression of the type I IFN-signaling pathway and induced the IL-10-associated M2-skewed immunosuppressive phenotype (54). This fact may deepen the degree of immunosuppression during the first stages of pregnancy and could adversely impact the immunological status of pregnant WLHIV, providing the microenvironment to support enhanced viral replication. Likewise, an expanded population of CD14+/CD16+ monocytes has been associated with higher viremia in individuals living with HIV (55). HIV RNA load is the most significant predictor of MTCT of HIV (34). In its turn, occurrence of the fetal deleterious effects of ZIKV infection could be facilitated by the resulting HIV immune activation and modification of the intrauterine cytokine environment as seen in the amniotic fluid of ZIKV-affected fetus (56).

It has also been established that ZIKV has placental tropism to various cells (57), The placenta could be reached by the ZIKV-infected maternal immune cells (58) or propagated from several types of cells in the maternal decidua to the cytotrophoblasts and fetal microvasculature. Also, ZIKV induces hyperplasia of Hofbauer cells, potentially intensifying the virus infection (59). Further, in the earliest phases of development, trophoblast cells are sensitive to ZIKV, and the virus could interfere in their differentiation (60). Trophoblast cells become increasingly resistant to ZIKV as the syncytium forms (61). As a consequence, the early placenta has a greater susceptibility to ZIKV infection in contrast to other microorganisms (51); this is concordant with evidence that vertical transmission of ZIKV occurs during the first trimester of maternal infection (16). In contrast, a substantial proportion of HIV transmission appears to occur around the time of delivery (62). Without intervention, the risk of HIV *in utero* transmission is about one-third (~7%) of the intrapartum risk demonstrating that innate and adaptive mechanisms restrict HIV infection within the placenta (63). However, the ZIKV infection in the placenta could promote stimulation of the placental Hofbauer cells, inflammation, thereby breaching in the syncytium trophoblasts layer and possibly promoting a local immunological environment that facilitates viral replication and dissemination of HIV across the placenta (64).

All these facts further emphasize the importance of studying the consequences of ZIKV infection in pregnant WLHIV. Therefore, we undertook this two-phase prospective cohort study to investigate both maternal and child effects of prenatal HIV and ZIKV co-infection. Delineation of possible harmful effects will allow formulation of standard-of-care recommendations to minimize adverse effects but enable continuation of preventive HIV therapy. Furthermore, while the prospective HIV ZIP study was developed before the COVID-19 pandemic, it is especially relevant today because the protocol design can be easily adapted to provide critically important information on the impact of COVID-19 infection or other still unrecognized new agents among pregnant women and their offspring worldwide.

## Ethics Statement

The studies involving human participants were reviewed and approved by Institutional Ethics Committees and the IRBs at the following institutions in Puerto Rico, mainland US, and Brazil: San Juan Hospital Research Unit, San Juan, Puerto Rico; University of Puerto Rico, San Juan, Puerto Rico; Children's Diagnostic & Treatment Center, Inc., Ft. Lauderdale, FL, USA; Bronx-Lebanon Hospital Center, Bronx, NY, USA; Baylor College of Medicine, Texas Children's Hospital, Houston, Texas, USA, University of Miami, Miami, Florida, USA; Instituto de Puericultura e Pediatria Martagão Gesteira, Federal University of Rio de Janeiro, Rio de Janeiro, Brazil; Hospital Federal dos Servidores do Estado, Rio de Janeiro, Brazil; Federal University of Minas Gerais, Belo Horizonte, Minas Gerais, Brazil; University of São Paulo, Ribeirão Preto, São Paulo, Brazil; and Hospital Geral de Nova Iguaçu, Nova Iguaçu, Rio de Janeiro, Brazil. Written informed consent to participate in this study was provided by the participants' legal guardian/next of kin.

## Author Contributions

Study design, development, and oversight were provided by the HIV ZIP Protocol Team (GS, MM-P, AA, NC, JM, KP, PW, BK, and PG). In addition, support in the following areas—study development and management, regulatory oversight, data management, laboratory, and technology and logistics—was provided by Westat, Rockville, MD (DM and FW). Additional support for study development was provided by RTI International, Research Triangle Park, NC (JL). All authors contributed to the article and approved the submitted version.

## Conflict of Interest

DM and FW were employed by the company Westat, Rockville, MD, United States. The remaining authors declare that the research was conducted in the absence of any commercial or financial relationships that could be construed as a potential conflict of interest.
