# Supplementary material for: An International Prospective Cohort Study of HIV and Zika in Infants and Pregnancy (HIV ZIP): Study Protocol
Source: Front Glob Womens Health. 2021 Jul 2;2:574327. doi: 10.3389/fgwh.2021.574327 (PMC8594009; doi:10.3389/fgwh.2021.574327)
Supplement: Supplementary file 1 [file Data_Sheet_1.DOCX]

# Appendix I

**Maternal Schedule of Evaluations for Asymptomatic and Symptomatic Women**

| **Evaluations / Procedures** | **All Participants** | **Enroll** | **Acute ZIKV-like Symptoms if**  **≥ 18 weeks**  **gestation** | **< 18 weeks gestation** | **Second Trimester 24 weeks gestation**  **(+/- 2 weeks)** | **Third Trimester 34 weeks gestation**  **(+/- 2 weeks)** | **Delivery (live birth or fetal loss)**  **(+ 48 hours)** | **6 weeks**  **postpartum (+/- 7 days)** | **On Study / Acute ZIKV- like Symptoms** |
| --- | --- | --- | --- | --- | --- | --- | --- | --- | --- |
|  | **Screening** |  | **Entry** | **Entry** | **All participants on study** | | | | |
|  | |  |  |  | | | | |  |
| Informed Consent | X |  | X | X |  |  |  |  |  |
| Basic demographics, last menstrual period, eligibility confirmation | X |  |  | X |  |  |  |  |  |
| Fetal ultrasound with fetal heartbeat | X |  | X | X | X | X | X |  | X |
| Provide/review Zika-symptom diary |  |  | X | X | X | X | X | X | X |
| Zika-like symptoms, health & medication history |  |  | X | X | X | X | X | X | X |
| Pregnancy questionnaire |  |  | X | X | X | X | X |  | X |
| Other risk factors questionnaire |  |  | X | X | X | X | X | X | X |
| Medical and medication history and results from clinical laboratory tests conducted for clinical care |  |  | X | X | X | X | X | X | X |
| Targeted Physical Exam |  |  | X | X | X | X | X | X | X |
| HIV-infected only: HIV viral load, absolute CD4/CD8  T-cells and % abstraction |  |  | X | X | X | X | X | X | X |
|  | |  | **LABORATORY EVALUATIONS**  **(Blood volumes in parentheses are collected only if indicated.)** | | | | | | |
| Urine or blood: βhCG pregnancy test | 1 ml |  |  |  |  |  |  |  |  |
| Blood:  ZIKV IgM Abs ZIKV RNA detection DENV IgM and IgG Abs, DENVNSI Ag  CHKV as needed | 6 ml |  |  | 6 ml | 6 ml | 6 ml | 6 ml | 6 ml | 6 ml |
| Urine: ZIKV RNA detection | X |  |  | X | X | X | X | X | X |
| Blood (HIV-infected only): HIV viral load |  |  | 3 ml | 3 ml | 3 ml | 3 ml | 3 ml | 3 ml | 3 ml |
| Blood (HIV-infected only): CD4/CD8 T-cells |  |  | 3 ml | 3 ml | 3 ml | 3 ml | 3 ml | 3 ml |  |
|  | |  | **BIOREPOSITORY SPECIMENS (if consented for future use)** | | | | | |  |
| Urine | X |  |  | X | X | X | X | X | X |
| Blood for plasma | 4 ml |  |  | 4 ml | 4 ml | 4 ml | 4 ml | 4 ml | 4 ml |
| Cord blood |  |  |  |  |  |  | 20 ml |  |  |
| Placental tissue |  |  |  |  |  |  | X |  |  |

# Appendix II

**Infant Schedule of Evaluations**

|  | **Birth**  **(+ 48 hours)** | **3 Months**  **(week 12 +/- 2 weeks)** | **6 Months**  **(week 24 +/- 2 weeks)** | **12 Months**  **(week 52 +/- 2 weeks)** |
| --- | --- | --- | --- | --- |
| Informed consent | X |  |  |  |
| Health history including laboratory tests results for clinical care | X | X | X | X |
| Physical Exam | X | X | X | X |
| Ophthalmologic exam | X (+4 weeks) | X (+/-4 weeks) | X (+/-4 weeks) | X (+/-4 weeks) |
| Neurological exam | X | X | X | X (+/-4 weeks) |
| Hearing assessment | X (+4 weeks) | X (+/-4 weeks) | X (+/-4 weeks) | X (+/-4 weeks) |
| HIV-related test results and treatments, if  applicable | X | X | X | X |
| **NEURODEVELOPMENTAL/NEUROPSYCHOLOGICAL EVALUATIONS** | | | | |
| Neurodevelopmental (ND) assessment for screening: ASQ-3^1^ or BSID-III^2^ Screening Test |  | X (-2weeks/+6 weeks) | X (-2weeks/+6 weeks) | X (-2weeks/+6 weeks) |
| ND assessment if failed ND screening: Complete BSID-III^2^ or referral for neurological assessment^3^ |  | X (-2weeks/+6 weeks) | X (-2weeks/+6 weeks) | X (-2weeks/+6 weeks) |
| **NEUROIMAGING RESULTS ABSTRACTION/ASSESSMENT** | | | | |
| Head Ultrasound | X | X | X | X |
| Head CT/MRI scan | X | X | X | X |
| **LABORATORY EVALUATIONS**  **(Blood volumes within parentheses are collected only if indicated.)** | | | | |
| Blood: ZIKV RNA detection ZIKV IgM Abs | 4 ml | 3ml | 3ml | 3ml |
| Urine: ZIKV RNA detection | X | X | X | X |
| **BIOREPOSITORY SPECIMENS (if consented for future use)** | | | | |
| Urine | X | X | X | X |
| Remnant Cerebrospinal Fluid | X | X | X | X |

*Notes: ^1^ASQ-3: Ages & Stages Questionnaire, 3^rd^, Edition (administered for Spanish language exposed infants only).*

*^2^BSID-III: Bayley Scales of Infant Development, 3^rd^ Edition (Screening Test, available in English and Portuguese; BSID-III Complete Test available in English, Spanish, and Portuguese).*

*^3^Infants who could not be assessed via ASQ-3 or BSID-III due to primary language exposure other than English, Spanish or Portuguese, or who failed the Complete BSID-III follow up evaluation due to ND screen failure, were referred to neurology.*
